# Effect of Extrinsic Pigmentation on Dimensional Stability, Hardness, Detail Reproduction, and Color of a Silicone

**DOI:** 10.1055/s-0042-1753458

**Published:** 2022-09-26

**Authors:** Júlio César Vieira Goiato, Victória Tiyemi Lopes, Clóvis Lamartine de Moraes Melo Neto, André Pinheiro de Magalhães Bertoz, Daniela Micheline dos Santos, Victor Augusto Alves Bento, Marcelo Coelho Goiato

**Affiliations:** 1 Department of Biomedicine, Unisalesiano, Araçatuba, São Paulo, Brazil; 2 Department of Dental Materials and Prosthodontics, São Paulo State University, School of Dentistry, Araçatuba, São Paulo, Brazil; 3 Department of Pediatric and Social Dentistry, School of Dentistry, São Paulo State University, Araçatuba, São Paulo, Brazil; 4 Oral Oncology Center, São Paulo State University, School of Dentistry, Araçatuba, São Paulo, Brazil

**Keywords:** maxillofacial prosthesis, silicone, pigmentation, coloring agents, hardness tests, color test

## Abstract

**Objective** The aim of the study is to evaluate the effect of extrinsic pigmentation on the dimensional stability, hardness, detail reproduction, and color of a silicone after thermocycling.

**Materials and Methods** Sixty samples of MDX4–4210 silicone (Dow Corning Corporation Medical Products) with intrinsic pink pigment (H-109-P, Factor II) and intrinsic opacifier (TiO) were fabricated. Two groups were created: Group 1—only intrinsic pigmentation (H-109P, Factor II + TiO) (Control); and Group 2—intrinsic (H-109P, Factor II + TiO) and extrinsic (Tan FE - 215, Factor II) pigmentation. The following tests were performed for each group: dimensional stability, Shore A hardness, detail reproduction, and color. Readings for the tests were taken before and after thermocycling (2,000 cycles). For dimensional stability and hardness, two-way analysis of variance (ANOVA) was used. One-way ANOVA was used for the color test. In case of significant statistical difference, the Tukey test was applied ( *p* <0.05). All samples achieved the same detail reproduction score, therefore, no statistical evaluation was performed.

**Results** For the dimensional stability test, comparing the initial time with the final time, there was a significant contraction in both groups after thermocycling. For the hardness test, comparing the time points, only group 1 showed a significant reduction in hardness after thermocycling. Groups 1 and 2 scored 2 for the detail reproduction test, before and after thermocycling. Comparing group 1 with group 2, there was no significant difference for color change.

**Conclusion** Based on the tests performed, extrinsic pigmentation did not show a negative effect on silicone, and therefore it can be indicated. The results of the dimensional, hardness, detail reproduction and color evaluations of the MDX4–4210 silicone were clinically acceptable in all cases in the groups with and without extrinsic pigmentation.

## Introduction

Literature studies report that silicone elastomer is the most used option for maxillofacial prostheses. 1 2 3 4 5 Factors such as high humidity and temperature variation can degrade a silicone. 1 5 Silicone prostheses can be in direct contact with human blood, saliva, sweat, water and food, as in situations of tongue and oronasal prostheses. 1 Thus, clinically, these prostheses are subject to temperature variations (caused by the patient's diet) and high humidity.

Maxillofacial prostheses usually need to be replaced within 1 year due to their color fading. 1 2 Despite this, it is not uncommon to observe patients wearing their prostheses for more than 1 year due to financial problems.

Other reasons to replace a silicone prosthesis include changes to its hardness and dimensions. The hardness of a silicone determines its flexibility. 2 A silicone prosthesis that simulates the flexibility of human skin promotes aesthetic and functional comfort to the patient. 2 The dimensional stability of the material of a prosthesis is important to maintain its adaptation to the place for which it was manufactured. 1 6 In addition, if a silicone prosthesis undergoes a significant dimensional change, it may lose its function and appearance. 1 6

A silicone prosthesis can be intrinsically pigmented. Despite this, in many situations it is important to use an extrinsic pigment to more accurately simulate the chromatic characteristics of human skin. 2 Thus, studies evaluating the effect of extrinsic pigmentation on the mechanical and physical properties of a silicone are important for literature.

A search was performed on PubMed using the keywords “pigment” and “extrinsic” and “silicone.” Only one article was found evaluating the effect of extrinsic pigmentation on the color and hardness of a silicone after accelerated aging. 2 Thus, the objective of the present study is to evaluate the effect of extrinsic pigmentation on the dimensional stability, hardness, detail reproduction, and color of a silicone after thermocycling.

## Materials and Methods

### Formation of Groups

Sixty samples of MDX4–4210 silicone (Dow Corning Corporation Medical Products, United States) with intrinsic pink pigment (H-109-P, Factor II, United States) and intrinsic opacifier (TiO) were fabricated.

Thirty samples were manufactured with dimensions of 30 mm(Ø) × 3 mm, 4 and another thirty were manufactured with dimensions of 22 mm (Ø) × 2 mm. 7 Thus, two groups were formed, and in each group there were 15 samples with dimensions of 30 mm (Ø) × 3 mm and 15 samples with dimensions of 22 mm (Ø) × 2 mm:

- Group 1: group with intrinsic pigmentation (H-109P, Factor II + TiO) and without extrinsic pigmentation (control).
- Group 2: group with intrinsic pigmentation (H-109P, Factor II + TiO) and extrinsic pigmentation (Tan FE - 215, Factor II, United States).

Using samples with dimensions of 30 mm (Ø) × 3 mm, dimensional stability, Shore A hardness, and detail reproduction tests were performed. 4 Samples with dimensions of 22 mm (Ø) × 2 mm were used for color evaluation. 7 Readings for these tests were taken before (initial) and after thermocycling (final).

### Sample Production

The silicone, intrinsic pigment, and intrinsic opacifier were weighed on a precision digital scale (BEL Analytical Equipment, Brazil). The intrinsic pigment and the opacifier each corresponded to 0.2% of the weight of the silicone. 1 2 The silicone was manipulated at 23° ± 2°C with a relative humidity of 50% ± 10%. 1 2 The two parts of silicone, Part A (catalyst) and Part B (base), were mixed until a homogeneous mass was obtained. Subsequently, intrinsic pigment and intrinsic opacifier were added to the silicone. For these steps, a vacuum mixture was performed using a mechanical spreader (Polidental, Brazil).

The pigmented silicone was inserted into two different metallic matrices, and a spatula was used to flatten and standardize its thickness. 1 The metal matrices were closed and subjected to a pressure of 1 ton for 10 minutes (Maxx II; Essence Dental VH, Brazil). 2 Samples remained confined in the matrices under a controlled temperature (29°C) with the surfaces exposed for 72 hours to complete the polymerization of the material. 1

The extrinsic pigment (Tan FE–215, Factor II) was diluted in 1,1,1-trichloroethane (I-301 Extrinsic Tri-Fluid, Factor II, United States) in the proportion of 1 mL (extrinsic pigment) to 1 mL (1,1,1-trichloroethane). 2 This extrinsic pigment diluted in 1,1,1-trichloroethane was uniformly blasted onto the surface of the samples (Group 2) with the help of an airbrush (WIMPEL, Brazil). 2 Subsequently, the extrinsic paint was sealed following the manufacturer's recommendations. The extrinsic pigment corresponded to 0.2% of the silicone weight. 2

### Dimensional Stability

A three-dimensional optical microscope (Quick Scope, Mitutoyo, United States) was used to calculate the dimensions of the samples. 8 This microscope had a digital table with a magnification of 350x and an accuracy of 1 μm. 8 Measurements were calculated using QSPAK software (Mitutoyo, United States). 8

### Hardness

The evaluation of the Shore A hardness test was performed with a digital durometer (GSD 709 Teclock, Japan) according to the American Society for Testing and Materials (Designation D2240). 2 The needle penetrated the samples at a load of 10 N. 2 The measurement was established between 0 and 100 Shore A with 1% of tolerance, and therefore hardness values were expressed in Shore A units. 2 Each sample was placed on the durometer table at a distance of  ± 2 mm from the penetration tip of the device. 2 The penetration tip applied pressure for 15 seconds on the samples. 2 Three measurements were performed on each sample, and later, an average of the three measurements was obtained. 2

### Detail Reproduction

In the detail reproduction test, the angular accuracy of three grooves (20 μm, 50 μm and 75 μm wide) molded in each sample was recorded. 4 Detail reproduction was examined using a stereo microscope (Olympus, Japan) under low-angle illumination at 13× magnification. 4 To classify the accuracy of detail reproduction, the scores suggested by Goiato et al were used as described below: X: no groove reproduction; 0: full reproduction of two of the three grooves; 1: full reproduction of the three grooves, with inaccurate angles; and 2: full reproduction of the three grooves, with accurate angles. 4

### Color Change

The color readings were taken using a spectrophotometer of visible ultraviolet reflection (UV-2450, Shimadzu, Japan). 2 Color alteration (ΔE) was calculated by the *Commission Internationale de L'Eclairage* (CIE) L*a*b* system. 2 The following formula was used: Δ *E* = [(Δ *L* ) ^2^ + (Δ *a* ) ^2^ + (Δ *b* ) ^2^ ] ^½^ . 2 The “ *L* ” represents brightness from 0 (black) to 100 (perfect white), the “ *a* ” represents the amount of red (positive values) or green (negative values), and the “ *b* ” represents the amount of yellow (positive values) or blue (negative values). 2

### Thermocycling

The samples were subjected to 2,000 immersion cycles in alternating 60-second baths of distilled water at 5 ± 1°C and 55 ± 1°C (MSCT-3, Convel, Brazil). 9 10

### Statistical Analysis

Statistical evaluations were performed using the Jamovi software (Version 2.2.5.0, Jamovi Project, Australia).

The interaction of pigment (presence or absence of extrinsic pigment) with dimensional stability or hardness was verified by two-way analysis of variance (ANOVA).

One-way ANOVA was used for the color test. In the case of statistical difference, the HSD Tukey test was applied.

In all cases, values were considered significant when *p* was less than 0.05.

All samples achieved the same detail reproduction score, so no statistical evaluation was performed.

## Results

For the dimensional stability test, there was a significant difference between time points (initial × final) for the groups with and without extrinsic pigmentation ( *p* <0.05) ( Table 1 ). Therefore, there was a significant contraction of samples from these groups after thermocycling.

**Table 1:** Mean results (%) of the dimensional stability test

Groups	Time points	
	Initial	Final
Group 1 (No extrinsic pigment)	0.89 Aa	1.26 Ab
Group 2 (With extrinsic pigment)	0.98 Aa	1.42 Ab

Note: (Tukey *p* <0.05) Different lowercase letters horizontally show statistical significance. Different uppercase letters vertically show statistical significance.

For the hardness test, only the group without extrinsic pigmentation showed a significant difference between time points ( *p* <0.05). Thus, there was a significant reduction in the Shore A hardness of this group after thermocycling ( *p* <0.05). Comparing group 1 with group 2 at the initial time point, the group without extrinsic pigmentation showed significantly greater Shore A hardness ( *p* <0.05) ( Table 2 ).

**Table 2:** Mean results of the Shore A hardness test

Groups	Time points	
	Initial	Final
Group 1 (no extrinsic pigment)	25.5 Aa	21.2 Ab
Group 2 (with extrinsic pigment)	21.4 Ba	22.7 Aa

Note: (Tukey *p* <0.05) Different lowercase letters horizontally show statistical significance. Different uppercase letters vertically show statistical significance.

Groups 1 and 2 scored 2 for the detail reproduction test, before and after thermocycling.

For the color test, comparing the group with extrinsic pigmentation (ΔE = 1.95) with the group without extrinsic pigmentation (ΔE = 1.70), there was no statistically significant difference ( *p* = 0.431).

## Discussion

Intrinsic and extrinsic factors can cause polymer degradation (e.g., silicone elastomer). 1 2 3 4 5 7 The intrinsic factor is related to changes in the silicone matrix, causing its degradation. 2 The extrinsic factors such as ultraviolet radiation, daily handling, temperature, air pollution, and high humidity can also cause degradation of this material. 2 5 In this study, thermocycling was used to simulate extreme conditions of high humidity as well as temperature changes (extrinsic factors) in the patient's mouth over a period of time. The literature reports that 2,000 thermal cycles clinically represent 2 years of wearing a complete denture, 9 10 11 so this could also represent 2 years of wearing a silicone prosthesis.

For the dimensional stability test, there was a significant contraction of samples in both groups after thermocycling, and this represents a degradation of the material ( Table 1 ). The highest contraction value observed in this study was 1.42% (Group 2). Despite this, the average dimensional change in the two groups, before and after thermocycling, remained within the standard recommended by ISO 4823, which states that the contraction should not exceed 1.5% after 24 hours (clinically acceptable). 4

For the extrinsic pigmentation group, there was no significant change in the Shore A hardness value after thermocycling ( Table 2 ). Probably, the extrinsic pigment acted as a protective factor for the hardness of the MDX4–4210 silicone during thermocycling, preventing the degradation of this physical property. It is possible to reach this conclusion because the group without extrinsic pigmentation showed a reduction in the hardness of the samples after thermocycling ( Table 2 ). Despite this, further studies are needed to confirm this result.

The ideal Shore A hardness should be 25 to 35 units or 25 to 55 units. 12 Despite this, it is possible to observe articles in the literature that found Shore A hardness values lower than 25 units for silicones after their production. 13 14 15 In addition, Goiato et al considered clinically acceptable the Shore A hardness of 18.08 units of a silicone. 14 Thus, based on this information, it is possible to consider all hardness values obtained in this study clinically acceptable ( Table 2 ).

Regarding the detail reproduction test, all groups scored 2. This score indicates that all samples had a full reproduction of the three grooves with accurate angles. This result is in agreement with Goiato et al, who reported that silicones have an excellent ability to reproduce details, reproducing grooves of up to 20μm wide. 16

Based on clinical acceptability threshold, Δ *E* <3.3 represents a clinically acceptable color change for a material, and Δ *E* ≥3.3 represents a clinically unacceptable color change for a material. 17 18 19 In the present study, the color changes observed in the evaluated groups were less than 3.3, and therefore, clinically acceptable.

In this study, an intrinsic opacifier (TiO) was used in all samples. This was done to simulate a clinical situation, as the opacifier prevents the silicone prosthesis from becoming translucent. In addition, this component has the function of protecting the silicone prosthesis from the chromatic changes caused by ultraviolet radiation. 20 TiO is used in the manufacture of sunscreens to protect human skin against ultraviolet rays, as it has a high refractive index. 21

Based on this study, the extrinsic pigment can be indicated for the manufacture of maxillofacial prostheses, as it does not cause disadvantages to silicone over time. It is also important to remember that the association between extrinsic and intrinsic pigmentation can more accurately mimic the chromatic characteristics of human skin. Despite the important results of the present study, more studies of this nature are needed.

## Conclusion

Based on the tests performed, extrinsic pigmentation did not show a negative effect on silicone, and therefore it can be indicated. The results of the dimensional, hardness, detail reproduction and color evaluations of the MDX4–4210 silicone were clinically acceptable in all cases in the groups with and without extrinsic pigmentation.
